# Song pattern recognition in crickets based on a delay-line and coincidence-detector mechanism

**DOI:** 10.1098/rspb.2017.0745

**Published:** 2017-05-24

**Authors:** Berthold Hedwig, Edith Julieta Sarmiento-Ponce

**Affiliations:** Department of Zoology, Cambridge University, Downing Street, Cambridge CB2 3EJ, UK

**Keywords:** phonotaxis, pattern recognition, delay-line, coincidence-detector

## Abstract

Acoustic communication requires filter mechanisms to process and recognize key features of the perceived signals. We analysed such a filter mechanism in field crickets (*Gryllus bimaculatus*), which communicate with species-specific repetitive patterns of sound pulses and chirps. A delay-line and coincidence-detection mechanism, in which each sound pulse has an impact on the processing of the following pulse, is implicated to underlie the recognition of the species-specific pulse pattern. Based on this concept, we hypothesized that altering the duration of a single pulse or inter-pulse interval in three-pulse chirps will lead to different behavioural responses. Phonotaxis was tested in female crickets walking on a trackball exposed to different sound paradigms. Changing the duration of either the first, second or third pulse of the chirps led to three different characteristic tuning curves. Long first pulses decreased the phonotactic response whereas phonotaxis remained strong when the third pulse was long. Chirps with three pulses of increasing duration of 5, 20 and 50 ms elicited phonotaxis, but the chirps were not attractive when played in reverse order. This demonstrates specific, pulse duration-dependent effects while sequences of pulses are processed. The data are in agreement with a mechanism in which processing of a sound pulse has an effect on the processing of the subsequent pulse, as outlined in the flow of activity in a delay-line and coincidence-detector circuit. Additionally our data reveal a substantial increase in the gain of phonotaxis, when the number of pulses of a chirp is increased from two to three.

## 1. Introduction

Signalling with repetitive sound patterns is an essential strategy for mate attraction in many insects and vertebrates [1–3]. Understanding how the animals process their communication signals in the auditory pathways and what specific mechanisms they employ to recognize mate-specific calls represent fundamental questions in neuroethology [4,5]. Owing to their simple song patterns some acoustically communicating insects are ideal systems to study auditory processing and feature detection. At the receiver side, auditory pattern recognition requires neural processing mechanisms tuned to the species-specific acoustic signals [6–8]. In female bispotted crickets (*Gryllus bimaculatus*), which orient to sequences of chirps composed of three to five sound pulses, behavioural studies have characterized the temporal tuning of phonotactic behaviour, which robustly represents the tuning of the underlying processing mechanism [9–12]. These studies also led to a concept of temporal pattern recognition based on a delay-line and a coincidence-detector [13,14]. According to this concept, the coincidence-detector integrates an internally delayed response to a sound pulse with the direct response to a subsequent pulse and responds best, when the pulse period matches the internal delay. As a fundamental principle of temporal processing, delay-lines and coincidence-detectors are also employed for the processing of pitch in the auditory system of mammals [15,16], for directional processing of sound signals in birds [17,18] and in visual pathways for the detection of movements [19]. In crickets, the auditory brain neurons of the pattern recognition network have recently been identified [14,20] with functional properties in close agreement with the delay-line and coincidence-detector concept.

An inherent consequence of this pattern recognition mechanism is that the coincidence-detector will receive a combination of different direct and delayed inputs for each sound pulse over the course of a chirp. Here, we test the hypothesis that manipulating the duration of individual sound pulses or pulse intervals at the beginning, middle and end of a chirp will have specific effects on cricket phonotaxis that reflect the mechanism of processing in the pattern recognition network.

## 2. Material and methods

### (a) Animals

Female last instar larvae of *Gryllus bimaculatus* were selected from a colony at the Department of Zoology, Cambridge University; they were individually housed, acoustically isolated from singing males, had continuous access to water and food, and were kept at 26–28°C. Phonotaxis tests were performed in a soundproof chamber and started 7–21 days after their final moult.

### (b) Trackball system

An open loop trackball system was used to measure the phonotaxis of tethered females walking towards sound patterns presented at 45° to the left or the right of their long axis. We calculated the lateral deviation towards the active speaker for the duration of each stimulus presentation. This provides a reliable measure of the phonotactic response (see [21,22] for details).

### (c) Acoustic patterns for phonotaxis tests

**Figure 1.**
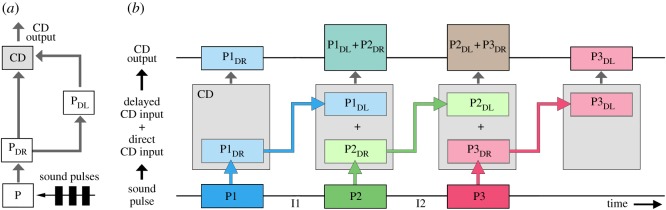
(*a*) Flow of activity within a delay-line coincidence-detector circuit. The response to a sound pulse (P) is forwarded directly (P_DR_) towards a coincidence-detector (CD) and also via a delay-line (P_DL_). If the internal delay matches the period of the pulse pattern, the direct spiking and the delayed graded input will coincide and the output of the detector is boosted. (*b*) Diagram revealing the flow of activity for a chirp with three sound pulses. Each sound pulse (P) elicits a direct (P_DR_) and a delayed input (P_DL_) to the coincidence-detector (CD). The CD output remains low (small boxes), if a direct input and a delayed input do not coincide, like the direct input by the first pulse (P1_DR_) or the delayed input by the third pulse (P3_DL_). When a direct and a delayed input coincide the CD output is boosted (large boxes), as for the delayed response to P1 coinciding with the direct response to P2 (P1_DL_ + P2_DR_).

We used chirps with three pulses and systematically changed the duration of either a single inter-pulse interval or a pulse, while keeping the duration of the other interval and of the pulses constant at 20 ms. The two inter-pulse intervals of a chirp are labelled I1 and I2 (figure 1*b*). In the interval paradigms, we adjusted I1 or I2 to 5, 10, 20, 25, 30, 40, 50, 60, 80 and 100 ms. The three pulses of a chirp are labelled as P1, P2 and P3 (figure 1*b*). In the pulse duration paradigm, P1, P2 or P3 were set to 5, 10, 20, 25, 30, 40, 50, 60, 80 and 100 ms. We also tested two chirp patterns. In one the duration of P1, P2 and P3 increased from 5 to 20 and 50 ms, and in the other reversed pattern it decreased correspondingly; inter-pulse intervals were 20 ms.

In all tests, we presented a sequence of two-pulse chirps with 20 ms pulse duration and 20 ms inter-pulse interval as a reference signal, which is the minimum chirp pattern that elicits a weak phonotactic response. This allowed us to report relative increases as well as decreases of the phonotactic response.

All sound patterns had a chirp period of 360 ms, pulses had a rising and falling ramp of 2 ms, except for 5 ms pulses where the ramp was 1 ms. The carrier frequency was 4.8 kHz and the sound intensity calibrated to 75 dB SPL_RMS_. Patterns with different pulse intervals or pulse durations were presented sequentially for 30 s from the left and right speaker, a silent period of 15 s separated different patterns to avoid any carry-over effects [22]. Tests were presented with increasing or decreasing order of pulse intervals or pulse durations. Each animal was tested three to five times with each paradigm.

### (d) Data analysis

The lateral deviation of a female towards the active speaker was measured for each test pattern over the course of 1 min combining the responses to the left and right presentation, and was averaged over all trials. As the behaviour of individuals varies (see [13]), we pooled data from 25 phonotactically responding females to obtain the characteristic response curves for changes in pulse intervals or durations. For each test, data for an example recording (*n* = 1) and the pooled results (*n* = 25) are listed in the electronic supplementary material, tables. Responses to test patterns are given with ±s.e.m. and are compared with the response to the two-pulse reference chirp, which was 14.9 ± 0.8 cm min^−1^. We describe the mean phonotactic responses as *strong*, *moderate*, *weak* or as *no-response*, depending on the significance levels by which responses were different from the reference response. Strong phonotaxis responses of more than 30 cm min^−1^ were always highly significantly different (*p* < 0.001) from the reference value, and also moderate responses in the range of 20–30 cm min^−1^ were different with high significance (*p* < 0.001). Phonotaxis responses with scores between 20 cm min^−1^ and 8.2 cm min^−1^ were not different from the reference value or were different at a significance level lower than *p* ≤ 0.003, and are described as weak responses. Any responses with scores lower than 8.1 cm min^−1^ had a significance level of *p* < 0.001 and are treated as no-response to the auditory pattern; further details are given in the text and electronic supplementary material. Calculations were performed in Excel (Microsoft Office Professional Plus 2013) and R (v. 3.2.5—Library Rcmdr). Shapiro–Wilk test (*p* < 0.05) confirmed that our data were not normally distributed; statistical analysis was performed using the Wilcoxon signed-rank test. For calculating the heat-map diagrams from the tuning curves (figure 4*e*), we linearly interpolated the phonotactic tuning curves at 5 ms intervals.

## 3. Results

The framework of our experiments is based on a delay-line and coincidence-detector mechanism for auditory pattern recognition (figure 1*a*) as proposed in [13]. In the brain, sound pulses (P) elicit a direct response (P_DR_) and, via a parallel line, an internally delayed response (P_DL_). Both are forwarded to a coincidence-detector (CD), which integrates the delayed response to a pulse P_DL_ with the direct response P_DR_ of a subsequent pulse. The CD requires a sequence of at least two pulses and is fully activated if the period of the pulse pattern corresponds to the internal delay. As an inherent property of this mechanism, the CD will receive different combinations of direct and delayed inputs at the beginning, middle and end of a chirp; this becomes obvious when the flow of activity is depicted (figure 1*b*).

### (a) Conceptual framework for the design of auditory test patterns

For a chirp with three sound pulses, the response to the first pulse (P1) will forward a direct input (P1_DR_) to the CD (figure 1*b*, left). The CD output will remain low (small box), as there is no previous pulse providing a delayed input. When the second pulse (P2) occurs, the delayed input P1_DL_ coincides with the direct input from this pulse (P2_DR_), and thus enhances the response of the CD (large box). Therefore, systematic changes of the duration of P1—while keeping all other parameters constant—are predicted to reveal the time course of the delayed input P1_DL_ to the CD. The effect should be mirrored by a characteristic change in phonotaxis.

Similarly, pulse P2 will generate a delayed input (P2_DL_), which will interact with the direct input from the next pulse (P3_DR_). Varying the duration of pulse P2 will have an impact on the CD output by interacting with the delayed response to P1 *and* by interacting with the direct input of P3 (figure 1*b*, middle), and should reveal to what degree the direct and/or the delayed signal of P2 shape the phonotactic response.

In the case of P3, the direct input (P3_DR_) will coincide with the delayed input P2_DL_ and enhance the CD output. Its delayed signal (P3_DL_), however, will not coincide with an input from a subsequent pulse and the CD will not be activated. Varying the duration of P3 will therefore demonstrate the effect of P3_DR_ on phonotaxis (figure 1*b*, right).

When keeping all pulse durations constant, the effect of inter-pulse intervals can be analysed. Changing inter-pulse interval I1 will alter the temporal overlap between the delayed input P1_DL_ and the direct input P2_DR_ at CD (figure 1*b*, left). As the direct input P2_DR_ is kept constant the time course by which P1_DL_ impacts on phonotaxis will be revealed. Similarly, varying interval I2 will change the temporal overlap between the delayed input P2_DL_ and the direct input P3_DR_, and will demonstrate how the delayed input P2_DL_ affects phonotaxis.

Based on this activity flow for chirps with three pulses, systematic changes in inter-pulse intervals and pulse durations are predicted to lead to characteristically different phonotactic responses. For each test, we present an example phonotaxis response from an individual female for a qualitative description and the pooled data from 25 phonotactically responding females.

### (b) Phonotactic responses to changes of pulse intervals I1 and I2

**Figure 2.**
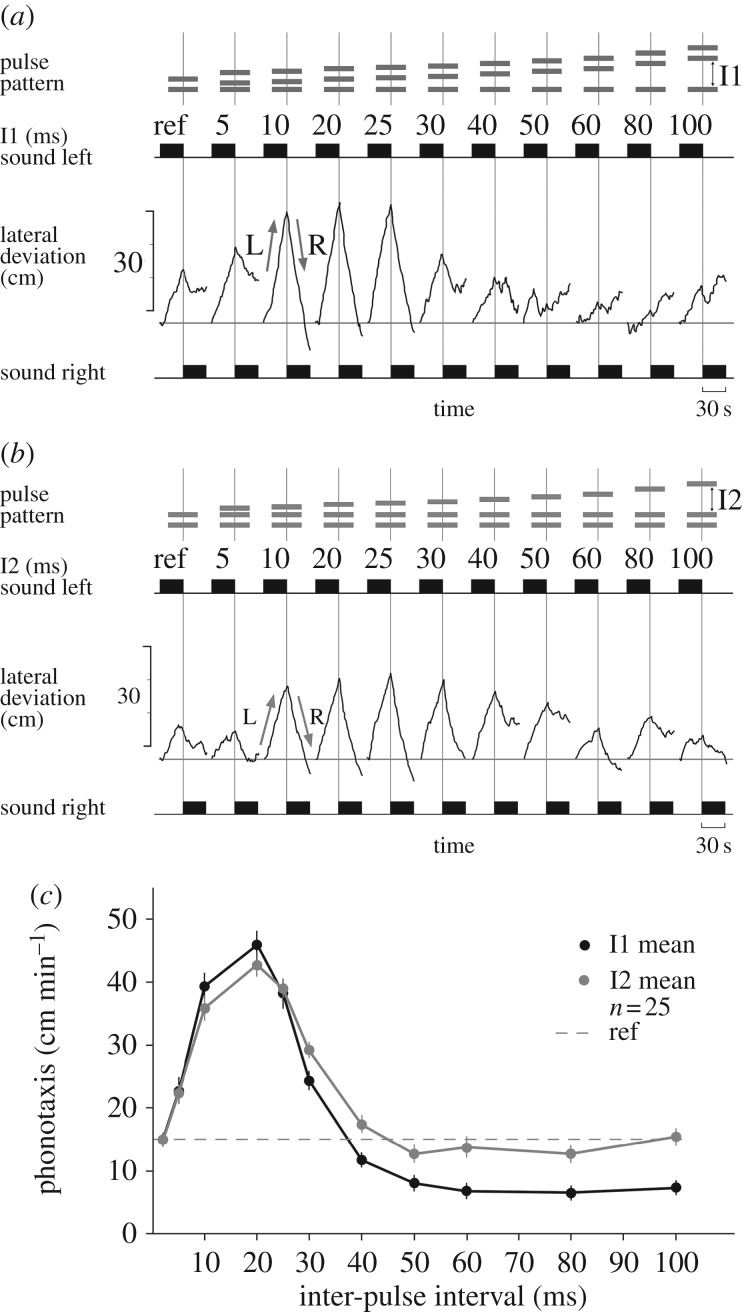
Effect of changing the duration of inter-pulse intervals on phonotaxis. (*a*) Chirp patterns with increasing duration of I1 (top); each pattern is presented as a sequence for 30 s from the left and right side (black rectangles). The lateral deviation (middle) indicates the phonotactic steering of a female towards the left (L, upwards) and then to the right (R, downwards) as the active speaker changes. The measurement is reset at the beginning of each test. (*b*) Chirp patterns with increasing duration of I2 (top) and phonotactic steering of a female towards 30 s sequences of chirp patterns with increasing duration of I2 (middle). (*c*) Characteristic responses for 25 females to changes in I1 (black line) and to changes in I2 (grey line). Responses are compared with the phonotactic response to a two-pulse reference chirp, indicated by a broken line. Mean responses are indicated by closed circles and connected by lines; for each chirp pattern the s.e.m. of the response is given.

The trackball measurements for testing the effect of changing the duration of I1 show a female's typical phonotactic steering response (figure 2*a*). Sound presentation for a test sequence always started with the left speaker; the lateral deviation measurement is reset to zero at the start of each test. During a strong phonotactic response (e.g. when I1 is at 10, 20 or 25 ms) the female first deviated towards the left speaker (L arrow) and when the active speaker changed, it walked to the right (R arrow), giving a combined steering response in the range of 73.6–75.8 cm min^−1^ for each test. For the reference chirp pattern and when I1 was 5, 30 or 40 ms, lateral deviation towards the active speaker was in the range of 22.6–30.1 cm min^−1^. For I1 of 50 to 100 ms, there was no directed response, phonotaxis failed and the steering signal became random. We pooled data over all females (figure 2*c*, black line) and compared the responses with the response to the reference two-pulse chirp of 14.9 ± 0.8 cm min^−1^, indicated by a dotted line. For I1 intervals of 10–25 ms, the phonotactic response was strong and highly significant larger than the reference value, with a maximum of 45.8 ± 2.2 cm min^−1^ at 20 ms. The response was moderate with 22.8 ± 2.0 cm min^−1^ at a 5 ms interval, and it was a weak interval of 40 ms with 11.7 ± 0.9 cm min^−1^. For I1 durations of 50 to 100 ms, the measurement was significantly lower than the reference value and in the range of 6.6 ± 0.7 to 8.1 ± 0.7 cm min^−1^ (figure 2*c*), indicating that long I1 led to a decrease and abandoning of phonotaxis.

The effect of changing interval I2 is presented for a female in figure 2*b*. A strong phonotactic response occurred at 10 to 30 ms with a combined lateral deviation towards the left and right active speaker in the range of 47.1–58.1 cm min^−1^. To the reference chirp and when I2 was 5 ms the phonotactic response was 14.1 cm min^−1^ and 14.4 cm min^−1^, respectively; even long I2 intervals of 50 to 100 ms duration elicited phonotactic steering in the range of 14.6 to 22.0 cm min^−1^. The pooled data reveal strong phonotaxis for I2 intervals between 10 and 30 ms, with a maximum response of 42.7 ± 1.9 at 20 ms (figure 2*c*, grey line). The response is moderate with 22.3 ± 1.5 cm min^−1^ at an interval of 5 ms. For long I2 durations of 40 to 100 ms, a weak phonotaxis response similar to the reference value was maintained in the range of 12.7 ± 6.6 to 17.4 ± 1.2 cm min^−1^.

For inter-pulse intervals of 5–40 ms, the tests demonstrate similar phonotaxis responses for both I1 and I2 with an optimum centred around 20 ms. Long I1 of 50–100 ms, however, abolished phonotaxis, whereas corresponding long I2 still elicited a weak response (figure 2*c*).

### (c) Effect of altering the duration of the first sound pulse (P1)

**Figure 3.**
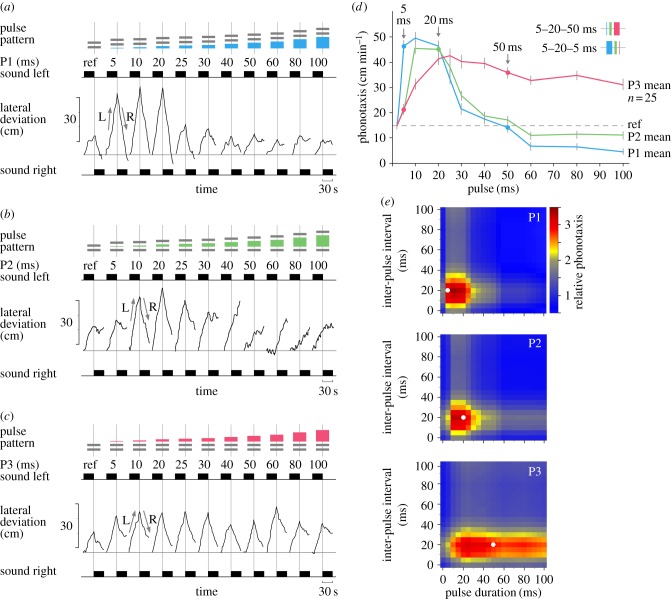
Effect of changing the duration of individual pulses on phonotaxis. (*a*) Chirp patterns with increasing duration of P1 (top, blue pulse) and phonotactic steering of a female towards sequences of chirp patterns with increasing duration of P1 (middle). (*b*) Chirp patterns with increasing duration of P2 (top, green pulse), and phonotactic steering of a female towards sequences of chirp patterns with increasing duration of P2 (middle). (*c*) Chirp patterns with increasing duration of P3 (top, red pulse), and phonotactic steering of a female towards chirp patterns with increasing duration of P3 (middle). (*d*) Characteristic tuning curves for changes of P1 (blue), P2 (green) and P3 (red). Further details as in figure 2. The tuning curves for P1, P2 and P3 indicate that chirps composed of 5, 20 and 50 ms pulses will be efficient to elicit phonotaxis; a lower response is expected if the chirps are played in reverse order. Selected pulses of the tuning curves are indicated and the chirp patterns with 5–20–50 ms and 50–20–5 ms pulses are given in the inset. (*e*) Heat maps of phonotactic responses calculated for the characteristic tuning curves of P1, P2 and P3, and the mean phonotactic response to I1 and I2. Blue colours indicate phonotaxis responses below and warm colours represent responses above the reference value, which is set to 1.0.

For testing the duration of pulse P1 (figure 3*a*, indicated in blue), the female provides an example of a very good tracker. Lateral deviation towards the reference chirp was 24.3 cm min^−1^ and for pulse durations of 5 to 20 ms, the lateral deviation even reached 84.1–90.8 cm min^−1^. The response then dropped from 51.6 to 20.9 cm min^−1^ for 25 to 40 ms pulses, and for pulse durations of 50–100 ms the orientation to the active speaker was 11.4–18.5 cm min^−1^ and less than the animal's reference response. For P1 durations of 5 to 25 ms, the pooled data (figure 3*d*, blue line) show a strong phonotactic response with 46.3 ± 2.3 cm min^−1^ at 5 ms, a maximum of 49.5 ± 2.2 cm min^−1^ at a P1 of 10 ms and a response of 33.8 ± 2.0 cm min^−1^ at 25 ms. The score then dropped towards a moderate response of 21.5 ± 1.9 at 30 ms, and a weak response of 17.6 ± 2.2 cm min^−1^ and 14.1 ± 1.9 cm min^−1^ at 40 and 50 ms, respectively. For P1 durations of 60–100 ms, the response data were significantly below the reference value and were only 4.6 ± 1.0 to 6.8 ± 1.3 cm min^−1^, indicating that test patterns with long P1 did not elicit phonotaxis.

### (d) Effect of altering the duration of the second pulse (P2)

When changes in the duration of P2 were tested (figure 3*b*, indicated in green), the steering of the cricket shown was 17.9 cm min^−1^ to the reference pattern and 27.6 cm min^−1^ when P2 was 5 ms. For P2 durations of 10 to 20 ms, the lateral deviation towards the active speaker increased and was in the range of 63.4–72.7 cm min^−1^. For P2 of 25 to 40 ms, it fell from 54.4 to 8.1 cm min^−1^, and between 50 ms and 100 ms the phonotactic response further decreased and was in the range of 7.7 cm min^−1^ to −13.1 cm min^−1^ as this female no longer showed a phonotactic response. When pooled over all females (figure 3*d*, green line), the phonotactic response for 5 ms was moderate and 21.3 ± 1.8 cm min^−1^. Phonotaxis was strong for P2 durations of 10 to 30 ms, and reached a broad maximum of 45.5 ± 1.7 cm min^−1^ at 10 ms and of 45.0 ± 2.3 cm min^−1^ at 20 ms. Between 40 ms and 100 ms weak phonotaxis occurred; the response dropped from 18.7 ± 1.6 cm min^−1^ to 11.0 ± 1.4 cm min^−1^, but was not highly significant different from the reference value.

### (e) Effect of altering the duration of the third pulse (P3)

When we changed the duration of P3 (figure 3*c*, indicated in red), the lateral deviation of the female walking towards the active speaker was 26.2 cm min^−1^ for the reference chirp, and for pulses of 5 to 30 ms it increased from 33.2 to −50.7 cm min^−1^. Phonotaxis still occurred for P3 durations of 40 to 100 ms as the lateral deviation was in the range of 36.3 to 26.1 cm min^−1^, respectively. The pooled data demonstrate that a 5 ms pulse lead to a weak phonotactic response of 21.6 ± 2.0 cm min^−1^ (figure 3*d*, red line). Phonotaxis increased for P3 durations of 10 to 25 ms and became strong; the maximum of the response curve is very broad with a peak of 42.6 ± 2.7 cm min^−1^ at 25 ms. Although the response then gradually dropped to a level of 31.3 ± 2.1 cm min^−1^ at P3 of 100 ms, overall strong phonotaxis was maintained for P3 durations of 10 to 100 ms as all test patterns caused a highly significant response of more than 30 cm min^−1^ and twice the reference value.

### (f) Comparison of characteristic tuning curves for changes in pulse durations

Different characteristic response curves were obtained for P1, P2 and P3 (figure 3*d*). A strong phonotaxis response of 46.3 cm min^−1^ was elicited by P1 at 5 ms, whereas P2 and P3 had to be at least 10 ms long to elicit a strong response. The maximum response changed for the three pulses; for P1 it was at 10 ms, for P2 it was in the range of 10–20 ms and for P3 it was at 25 ms. Long P1 of 60–100 ms had a negative effect and abolished phonotaxis, long P2 elicited a weak phonotaxis response, whereas long P3 elicited a strong response. We pooled the tuning curves for both pulse intervals and combined these with the curves for the pulse durations to generate colour-coded heat maps representing the strength of the phonotaxis response (figure 3*e*). The maps for P1 (top) and P2 (middle) show a similar pattern, with the centre of the P1 response shifted towards 5 ms pulse durations; the map for P3 (bottom) stands out as its maximum is shifted towards longer pulses and as it reveals sustained phonotaxis even towards long sound pulses. The characteristic tuning curves and the heat maps reveal the impact of pulse and interval durations on phonotaxis, and indicate that the auditory pattern recognition system is in a different functional state for each pulse that is processed during a chirp.

### (g) Designing and testing putative attractive and non-attractive chirp patterns

The different tuning curves for P1, P2 and P3 prompted us to explore the underlying processing mechanism in more detail. The comparison of the three tuning curves (figure 3*d*) with the reference response shows that a 5 ms pulse strongly enhanced the gain of phonotaxis by a factor of 3.1 when presented as P1, but when presented as P3 it only has a moderate effect of 1.4. A pulse of 20 ms duration always had a strong effect when presented either as P1, P2 or P3. A 50 ms pulse had a weak effect of 0.9 on the phonotactic response when presented as P1; however, when presented as P3 it strongly enhanced phonotaxis by a factor of 2.4. Consequently, chirps combining a 5 ms, a 20 ms and a 50 ms pulse (5–20–50 ms) should be effective to elicit phonotaxis. However, when played in reverse order (50–20–5 ms) they should be much less attractive. Presenting crickets with these patterns composed of the same sound pulses, just in reverse order (figure 3*d* inset), should reveal if these patterns have a specific impact on the processing of chirps, based on pulse duration and arrangement.

**Figure 4.**
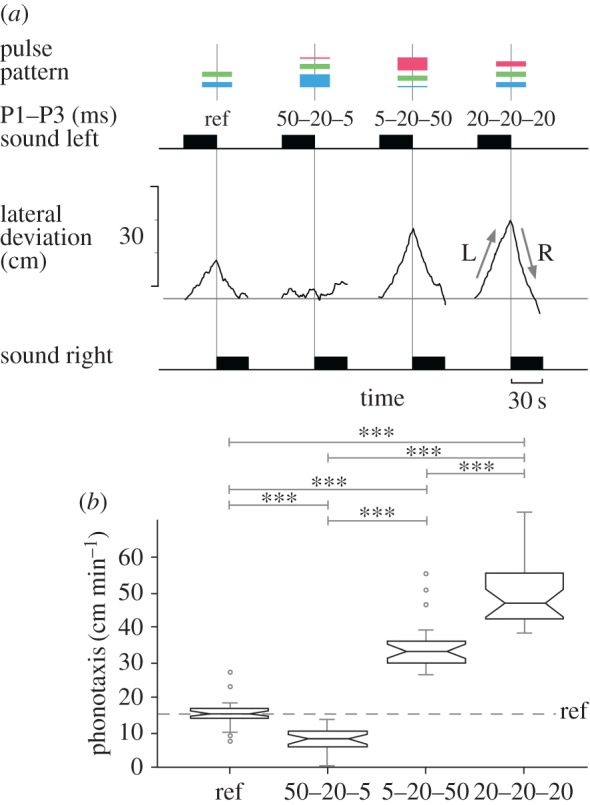
(*a*) Phonotactic steering of a female towards the reference chirp with 20–20 ms pulses, to a chirp pattern with 50–20–5 ms pulses, to chirps with 5–20–50 ms pulses and to chirps with 20–20–20 ms pulses. (*b*) Phonotaxis score of 25 females, presented as box-and-whisker plots; whiskers represent the 25th and 75th percentile (lower and upper quartiles, respectively), the band in the middle of the box is the 50th percentile (the median) and open circles indicate outliers. Significance levels for differences between the responses are indicated at the top of the diagram, ***p < 0.001. (Online version in colour.)

In these tests, we presented a sequence of the reference chirp with 20–20 ms pulses, the putative non-attractive chirp with 50–20–5 ms pulses, the putative attractive chirp with 5–20–50 ms pulses and also a chirp with 20–20–20 ms pulses, which in the previous tests elicited a strong phonotaxis response in the range of 41.4–46.5 cm min^−1^. Pulse intervals were kept at 20 ms (figure 4*a*). The recordings of a walking female show a lateral deviation of 27.0 cm min^−1^ to the reference chirp pattern, the lateral deviation to the 50–20–5 ms pulse pattern is 3.0 cm min^−1^, lateral deviation is 46.1 cm min^−1^ to the 5–20–50 ms pulse pattern and it is 47.6 cm min^−1^ to the 20–20–20 ms chirps. When pooled for all females (figure 4*b*), phonotaxis towards the 50–20–5 ms pattern was 7.9 ± 0.8 cm min^−1^ and significantly lower than the reference of 14.9 ± 0.8 cm min^−1^ (*p* < 0.001). The 5–20–50 ms chirps elicited a strong phonotaxis response of 34.7 ± 1.4 cm min^−1^, which was significantly higher than the reference value (*p* < 0.001) and significantly higher than the value of the 50–20–5 ms pattern (*p* < 0.001). The phonotaxis response towards the 20–20–20 ms chirp pattern reached 48.8 ± 1.9 cm min^−1^ and was significantly higher than any other response (*p* < 0.001). The very different phonotactic responses towards the attractive 5–20–50 ms and the non-attractive 50–20–5 ms chirps confirm that the functional state of the pattern recognition system depends on the duration of the pulses that are processed, and that it changes during the course of a chirp.

## 4. Discussion

### (a) Comparison to previous phonotaxis experiments

Cricket phonotaxis experiments were typically performed by altering the duration of all pulses or pulse intervals in a chirp [9–12,23]. In *G. bimaculatus* and its sister species *G. campestris*, the resulting tuning curves [9–11,20,24] point to the importance of pulses and inter-pulse intervals of 15–25 ms for calling song pattern recognition, and show that short (5 ms) and long (50 ms) pulses and pulse intervals are not efficient [9–11,20]. Owing to the previous design of these test paradigms, the phonotactic response always depended on changes in all pulses and/or pulse intervals of a chirp pattern. Here we analysed cricket phonotaxis in response to chirp patterns in which the duration of one pulse or one pulse interval was systematically altered. This allowed us to single out specific effects on the behaviour, which now can be linked to the neuronal processing underlying song pattern recognition, as so far revealed by single cell recordings [14,20,25].

### (b) Phonotactic tuning curves and neural processing in the delay-line coincidence-detector circuit

**Figure 5.**
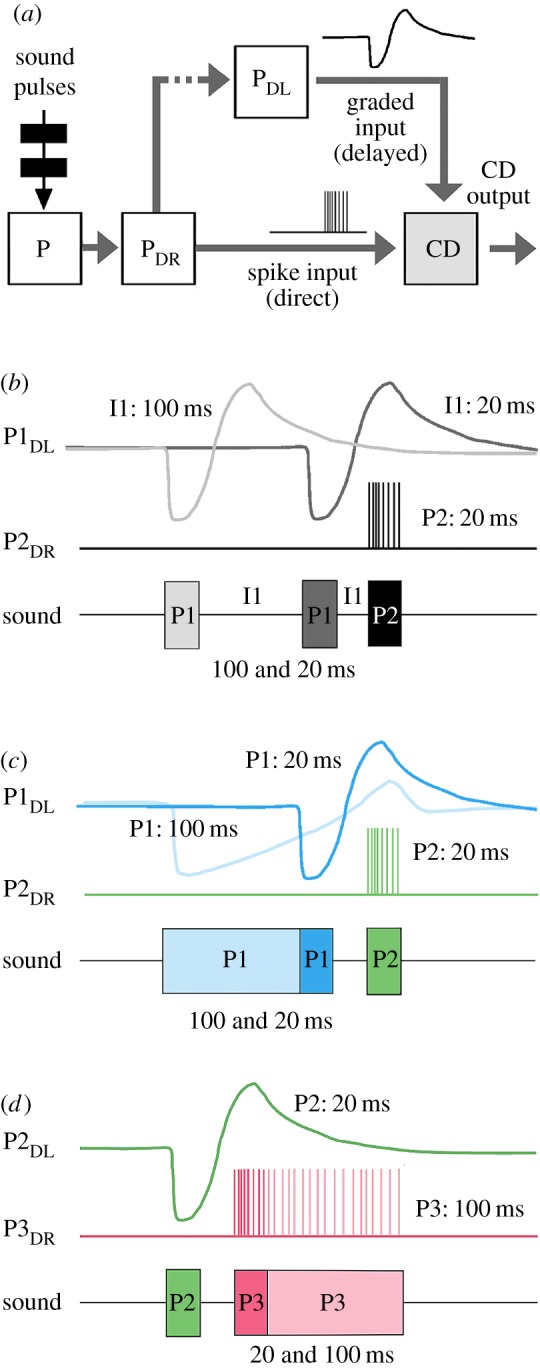
(*a*) Diagram indicating the flow of neuronal activity in the delay-line and coincidence-detector circuit in response to a sound pulse (P). Spike activity provides the direct input (P_DR_) to the CD, whereas the delayed input (P_DL_) is based on a graded excitatory signal following an initial inhibitory response due to post-inhibitory rebound. (*b*) Interaction of the direct and delayed input to CD when the duration of interval I1 is altered. For 20 ms I1 (dark grey symbols) the direct spiking response to P2 coincides with the peak of the graded excitation. For 100 ms I1 (light grey symbols) the P2 spikes coincide only with the falling phase of the graded signal. (*c*) Interaction of the direct and delayed input to CD when the duration of P1 is altered. For 20 ms P1 (dark blue symbols), the P2 spikes coincide with the full blown graded excitation. For 100 ms P1 (light blue symbols), only a weak delayed excitation coincides with the spike response to P2. (*d*) Interaction of the delayed and direct input to CD when the duration of pulse P3 is altered. For short 20 ms P3 (dark red symbol), the spike response coincides with the peak of the graded response to P2. Even for long 100 ms P3 the initial part of the spike response coincides with the peak of the graded excitation but the subsequent part of the spike response (light red symbol) only coincides with the decaying graded signal. In (*c,d*) the 100 ms pulse spans both parts of the blue/red sound stimulus symbol. For clarity, latencies are not considered in the diagram.

A previous concept [13] and intracellular studies of auditory brain neurons point to a delay-line and coincidence-detector circuit for pattern recognition in the cricket brain [14]. Sound pulses (P) elicit a direct response (P_DR_) mediated by the activity of a spiking ascending interneuron, and via a parallel line an internally delayed response (P_DL_). The delayed response is a graded excitatory potential (figure 5*a*) of a non-spiking neuron, which is generated after an initial inhibition of the interneuron. The direct and the delayed signals are forwarded to a coincidence-detector (CD), which integrates the delayed response to a pulse P_DL_ with the direct response P_DR_ of a subsequent pulse. Its response is strongest at the species-specific pulse-period, when the spiking response coincides with the delayed excitatory graded response. The interaction of the graded and spiking response is outlined for intervals and pulses of 20 and 100 ms (figure 5*b–d*).

Changing interval I1 alters the temporal interaction between the delayed input P1_DL_ and the direct input P2_DR_ at CD (figure 1*b*), and reveals the time course by which the graded signal P1_DL_ impacts on phonotaxis. Correspondingly varying interval I2 demonstrates the effect of P2_DL_. Based on the neuronal processing, both characteristic response curves reveal how the amplitude of the excitatory delayed signal changes with the duration of the pulse interval (figure 5*b*). For interval durations of 20 ms, the spike activity of P2_DR_ coincides with the graded excitatory response (figure 5*b*, dark grey line) and will activate the CD. I1 and I2 showed a similar tuning curve with a best response at 20 ms (figure 2*c*). These phonotactic responses to changes in pulse intervals are in good agreement with the time course of the graded delayed response recorded in the pattern recognition network in the brain [14, fig. 5*b*]. They also correspond to previously reported tuning curves [20]. At intervals of 100 ms, the spiking activity coincides only with the falling phase of the delayed graded signal (figure 5*b*, bright grey line) and the CD will not be activated. I1 intervals longer than 50 ms abolished phonotaxis; the nature of this effect is not yet clear. Negative effects on phonotaxis due to non-attractive signals have also been reported before [26]. In the case of I2, however, weak phonotaxis is maintained as processing of the first two pulses of the chirps is not affected by changes of I2.

Corresponding to the flow of activity in the circuit, the P1 tuning curve (figure 3*d*) reflects how the time course and amplitude of the graded delayed signal P1_DL_ depend on the duration of P1. For 20 ms P1 pulses, the maximum of the graded signal (figure 5*c*, dark blue line) will coincide with the direct spiking input of P2 and will drive the CD response, whereas for 100 ms durations of P1 spike activity will coincide only with a weak excitatory delayed signal (figure 5*c*, light blue line). Different from previous data on pattern recognition [9–11,20], pulse durations of 5 ms were efficient to elicit strong phonotaxis and the best response occurred at 10 ms. In electrophysiological experiments 5 ms pulses have not yet been tested, but pulses longer than 10 ms were sufficient to trigger the graded response in the delay-line neuron. Interestingly, in electrophysiological experiments the graded delayed response was always coupled to the end of a sound pulse, even for 50 ms long pulses [14, fig. 4*d*]. Also, the coincidence-detection mechanism tolerates 50 ms pulses [25, fig. 7*b*]. We therefore expected that phonotaxis would not be affected when the duration of P1 was increased to 50 ms and beyond. The tuning curve for P1 (figure 3*d*), however, shows that this is not the case, as phonotaxis reliably started to break down for P1 longer than 30 ms.

Pulse P2 generates a delayed input (P2_DL_), which interacts with the direct input from the next pulse (P3_DR_). Varying the duration of pulse P2 therefore has an impact on the CD by interacting with the delayed response to P1 *and* by interacting with the direct input of P3 (figure 1*b*, middle); thus the direct and/or the delayed signal of P2 may shape the phonotactic response. The tuning curve for P2 (figure 3*d*) shows the best response at a 20 ms pulse duration, as previously reported for standard chirps [9–11,20]. It is similar to the tuning curve obtained by changing I2 (figure 2*d*) and the P1 tuning curve (figure 3*d*), indicating that the delayed graded response of P2 provides the dominant effect on phonotaxis.

In the case of P3, the direct spiking input (P3_DR_) coincides with the graded delayed excitatory input P2_DL._ The delayed signal P3_DL_, however, does not coincide with any subsequent signal (figure 1*b*, right). Varying the duration of P3 therefore demonstrates the effect of P3_DR_ on phonotaxis. The characteristic curve (figure 3*d*) shows an increase of the response up to 25 ms, which is in line with previous data for standard chirps [9–11,20] and the time course of the delayed graded excitation of the delay-line neuron [14]. However, different from previous data, P3 durations up to 100 ms were efficient to drive phonotaxis. This may be elucidated by the interaction of the direct spike response P3_DR_ with the delayed graded response P2_DL_ (figure 5*d*). For P3 pulses of 20 ms duration, the spike activity (figure 5*d*, dark red spikes) will coincide with the peak of the graded excitatory response driven by P2. Even for 100 ms pulses, the initial spike response (P3_DR_) will coincide with the peak of the graded signal P2_DL._ This will boost the response of the CD, and the phonotactic response even to long P3 may be explained. The ongoing spike response of P3_DR_ (figure 5*d*, light red spikes) will only coincide with the decaying part of the graded signal and will not enhance the CD output. As the delayed signal P3_DL_ will not coincide with any subsequent signal (figure 1*b* right), this part of the response has no effect on phonotaxis.

### (c) A pattern recognition concept based on a delay-line and coincidence-detector mechanism

Testing the pulse duration of P1, P2 or P3 revealed three different characteristic responses (figure 3*d,e*). From P1 to P3, there is a shift of the best response towards longer pulse durations. For P1, the response is tuned towards 5–25 ms with a peak at 10 ms, the response to P2 is best at 10–30 ms with a broad peak at 10–20 ms, and for P3 the range is 10–100 ms with a peak at 25 ms. Long P1 pulses of 60–100 ms abolished phonotaxis, in the case of P2 weak phonotaxis was maintained, and long P3 elicited a strong response. In different crickets, bush-crickets and grasshoppers such different tuning curves may be described as species-specific profiles for acoustic communication [27]. In *G. bimaculatus*, however, such different filter properties occur sequentially while the pulses of chirps are processed.

Short (5 ms) or long (50 ms) pulses have very different effects on phonotaxis, when the pulses are presented at the beginning or the end of a chirp (figure 3*d*,*e*). Correspondingly, a chirp pattern composed of 5–20–50 ms pulses elicited phonotaxis, but failed to do so when played in reverse order of 50–20–5 ms (figure 4). Changes in the responsiveness to reversely played song patterns have not yet been reported in crickets but do occur in grasshoppers [28]. In crickets, the different responses towards these chirps with increasing or decreasing pulse durations reveal that the functional state of the processing mechanism changes while sequences of sound pulses are processed. These changes depend on the duration of pulses and intervals, and determine the pattern-recognition process. The characteristic tuning curves provide a look-up table to compose attractive and non-attractive chirp patterns.

The characteristic curves do not indicate a constant, steady filter process underlying song pattern recognition, but rather that filtering is based on dynamic changes in the processing properties of the neuronal network. These dynamic changes can be linked to the neuronal activity within the delay-line and coincidence-detector circuit in the cricket brain [13,14,25], in which the response to a sound pulse impacts on the processing of the subsequent pulse.

### (d) Changes in the gain of the phonotactic response

In comparison with the response to the two-pulse reference chirp, a three-pulse chirp increased the phonotactic response by a factor of more than 3.0, indicating a nonlinear impact on the gain of the response. An increase of the response by a factor of 1.5 might be expected if the score simply scales with the number of pulses in a chirp and an increase by a factor of 2.0 as the coincidence detector is fully activated twice due to the additional pulse. Moreover, first pulses (P1), which were followed by a long interval (I1) or which had a long duration, decreased the gain and abolished phonotaxis. These nonlinear changes of the response are not predicted by the mechanistic temporal processing in the delay-line and coincidence-detector. They rather indicate an additional facilitation and/or neuromodulation effect that changes auditory processing and that currently is not reflected in the response properties of the auditory brain neurons [14]. As the change in gain has an impact on the processing of subsequent sound pulses, its rather long time course may provide a filter at a different time scale as required for the processing of chirp durations [11,26].

## 5. Conclusion

Our analysis demonstrates how detailed phonotaxis responses can be revealed and linked to the organization of auditory pattern recognition based on a delay-line and coincidence-detector mechanism [13,14]. Further electrophysiological experiments should elucidate how the characteristic tuning curves are mirrored in the neuronal activity of the network. Recent computational approaches to auditory pattern recognition in insects based on Gabor filters present a phenomenological description of the phonotaxis preference functions [24,29,30]. The current experiments provide the basis to refine computational approaches to model this pattern-recognition system. They also may inspire behavioural experiments to analyse delay-line and coincidence-detection mechanisms in other sensory pathways.

## Supplementary Material

Song pattern recognition in crickets based on a delay-line and coincidence-detector mechanism

## References

[1] Vehrencamp S, Bradbury J 1998 Principles of animal communication. Sunderland, MA: Sinauer Associates.

[2] Gerhardt HC, Huber F 2002 Acoustic communication in insects and anurans: common problems and diverse solutions. Chicago, IL: University of Chicago Press.

[3] Hedwig B (ed.). 2014 Insect hearing and acoustic communication: animal signals and communication, *vol 1*. Berlin, Germany: Springer.

[4] Bullock TH 1961 The problem of recognition in an analyzer made of neurons. In Sensory communication (ed. WA Rosenblith), pp. 717–724. Cambridge, MA: MIT Press.

[5] Martin KAC 1994 A brief history of the ‘feature detector’. Cereb. Cortex 4, 1–7. (10.1093/cercor/4.1.1) 8180488

[6] Hoy RR 1978 Acoustic communication in crickets: a model system for the study of feature detection. Fed. Proc. 37, 2316–2323. 354967

[7] Von Helversen O, Von Helversen D 1987 Innate receiver mechanisms in the acoustic communication of Orthopteran insects. In Aims and methods in neuroethology (ed. Guthrie DM), pp. 104–150. Manchester, UK: Manchester University Press.

[8] Hedwig B 2016 Sequential filtering processes shape feature detection in crickets: a framework for song pattern recognition. Front. Physiol. 7, 1–15. (10.3389/fphys.2016.00046) 26941647 PMC4766296

[9] Thorson J, Weber T, Huber F 1982 Auditory behavior of the cricket. II. Simplicity of calling-song recognition in *Gryllus*, and anomalous phonotaxis at abnormal carrier frequencies. J. Comp. Physiol. A 146, 361–378. (10.1007/BF00612706)

[10] Doherty JA 1985 Temperature coupling and ‘trade-off’ phenomena in the acoustic communication system of the cricket, *Gryllus bimaculatus* De Geer (Gryllidae). J. Exp. Biol. 114, 17–35.

[11] Doherty JA 1985 Trade-off phenomena in calling song recognition and phonotaxis in the cricket, *Gryllus bimaculatus* (Orthoptera, Gryllidae). J. Comp. Physiol. A 156, 787–801. (10.1007/BF00610831)

[12] Hedwig B 2006 Pulses, patterns and paths: neurobiology of acoustic behaviour in crickets. J. Comp. Physiol. A 192, 677–689. (10.1007/s00359-006-0115-8) 16523340

[13] Weber T, Thorson J 1989 Phonotactic behavior of walking crickets. In Cricket behavior and neurobiology (eds Huber F, Moore TE, Loher W), pp. 310–339. Ithaca, NY: Cornell University Press.

[14] Schöneich S, Kostarakos K, Hedwig B 2015 An auditory feature detection circuit for sound pattern recognition. Sci. Adv. 1, 1–7. (10.1126/sciadv.1500325) PMC4643773 26601259

[15] Langner G 1997 Neural processing and representation of periodicity pitch. Acta Otolaryngol. 117, 68–76. (10.3109/00016489709126147) 9442847

[16] Langner G 1997 Temporal processing of pitch in the auditory system. J. New Music Res. 26, 116–132. (10.1080/09298219708570721)

[17] Carr CE 1993 Delay line models of sound localization in the barn owl. Am. Zool. Soc. Integr. Comp. Biol. 33, 79–85.

[18] Grothe B 2003 New roles for synaptic inhibition in sound localization. Nat. Rev. Neurosci. 4, 540–550. (10.1038/nrn1136) 12838329

[19] Reichardt W 1987 Evaluation of optical motion information by movement detectors. J. Comp. Physiol. A 161, 533–547. (10.1007/BF00603660) 3681769

[20] Kostarakos K, Hedwig B 2012 Calling song recognition in female crickets: temporal tuning of identified brain neurons matches behavior. J. Neurosci. 32, 9601–9612. (10.1523/JNEUROSCI.1170-12.2012) 22787046 PMC6622257

[21] Hedwig B, Poulet JFA 2004 Complex auditory behaviour emerges from simple reactive steering. Nature 430, 781–785. (10.1038/nature02787) 15306810

[22] Hedwig B, Poulet JFA 2005 Mechanisms underlying phonotactic steering in the cricket *Gryllus bimaculatus* revealed with a fast trackball system. J. Exp. Biol. 208, 915–927. (10.1242/jeb.01452) 15755890

[23] Hennig RM 2009 Walking in Fourier's space: algorithms for the computation of periodicities in song patterns by the cricket *Gryllus bimaculatus*. J. Comp. Physiol. A 195, 971–987. (10.1007/s00359-009-0473-0) 19756649

[24] Hennig RM, Heller K-G, Clemens J 2014 Time and timing in the acoustic recognition system of crickets. Front. Physiol. 5, 286 (10.3389/fphys.2014.00286) 25161622 PMC4130308

[25] Kostarakos K, Hedwig B 2015 Pattern recognition in field crickets: concepts and neural evidence. J. Comp. Physiol. A 201, 73–85. (10.1007/s00359-014-0949-4) 25348550

[26] Grobe B, Rothbart MM, Hanschke A, Hennig RM 2012 Auditory processing at two time scales by the cricket *Gryllus bimaculatus*. J. Exp. Biol. 215, 1681–1690. (10.1242/jeb.065466) 22539735

[27] Hennig RM, Franz A, Stumpner A 2004 Processing of auditory information in insects. Microsc. Res. Tech. 63, 351–374. (10.1002/jemt.20052) 15252878

[28] Von Helversen D, Von Helversen O 1998 Acoustic pattern recognition in a grasshopper: processing in the time or frequency domain? Biol. Cybern. 79, 467–476. (10.1007/s004220050496)

[29] Clemens J, Hennig RM 2013 Computational principles underlying the recognition of acoustic signals in insects. J. Comput. Neurosci. 35, 75–85. (10.1007/s10827-013-0441-0) 23417450

[30] Ronacher B, Hennig RM, Clemens J 2015 Computational principles underlying recognition of acoustic signals in grasshoppers and crickets. J. Comp. Physiol. A 201, 61–71. (10.1007/s00359-014-0946-7) 25258206

