# Supplementary material for: Song pattern recognition in crickets based on a delay-line and coincidence-detector mechanism
Source: Proc Biol Sci. 2017 May 24;284(1855):20170745. doi: 10.1098/rspb.2017.0745 (PMC5454277; doi:10.1098/rspb.2017.0745)

**Proceedings of the Royal Society B**

Proc. R. Soc. B 20170745. <http://dx.doi.org/10.1098/rspb.2017.0745>

**Song pattern recognition in crickets based on a delay-line and coincidence-detector mechanism**

Berthold Hedwig* and Edith Julieta Sarmiento-Ponce

Department of Zoology, Downing Street, CB2 3EJ Cambridge, UK

*E-mail: [bh202@cam.ac.uk](mailto:bh202@cam.ac.uk)

Phone: 0044 1223 336603

# Supplementary tables listing data of an example response and mean data for all phonotactic test

## Data for Interval I1

Table I1. Statistical summary for I1, giving an exemplary single trial response (Expl.), the mean and median for all animals tested (n=25); and Wilcoxon Signed Rank test comparing the I1 vs Ref. Bold data are highly significantly different from the reference value.

|  | **Expl.** | **Mean**  **n=25** | **Median**  **n=25** | **SD** | **SEM** | **Min** | **1^st^ Qu.** | **3^rd^ Qu.** | **Max** | **p-value** |
| --- | --- | --- | --- | --- | --- | --- | --- | --- | --- | --- |
| **Ref** | 22.6 | 14.9 | 15.1 | 4.2 | 0.8 | 7.3 | 13.9 | 16.7 | 27.1 | - |
| **5** | 30.1 | 22.8 | 22.7 | 10.1 | 2.0 | 2.9 | 18.4 | 26.6 | 52.2 | **0.001** |
| **10** | 73.9 | 39.3 | 40.7 | 9.9 | 2.0 | 20.5 | 34.7 | 45.2 | 59.6 | **0.001** |
| **20** | 75.8 | 45.8 | 45.5 | 11.1 | 2.2 | 26.4 | 44.1 | 47.9 | 76.7 | **0.001** |
| **25** | 73.6 | 38.1 | 38.8 | 11.5 | 2.3 | 11.4 | 32.7 | 41.6 | 78.4 | **0.001** |
| **30** | 29.0 | 24.3 | 24.0 | 7.1 | 1.4 | 11.7 | 20.4 | 26.0 | 50.4 | **0.001** |
| **40** | 24.6 | 11.7 | 11.5 | 4.3 | 0.9 | 3.3 | 9.1 | 12.6 | 24.0 | **0.004** |
| **50** | 0.0 | 8.1 | 8.0 | 3.5 | 0.7 | 0.3 | 6.6 | 10.0 | 15.3 | **0.001** |
| **60** | 3.2 | 6.8 | 6.4 | 3.8 | 0.8 | 0.0 | 5.3 | 8.4 | 16.5 | **0.001** |
| **80** | -1.1 | 6.6 | 6.4 | 3.3 | 0.7 | 0.3 | 5.3 | 7.7 | 17.1 | **0.001** |
| **100** | 6.3 | 7.3 | 7.8 | 3.1 | 0.6 | 0.6 | 5.8 | 8.8 | 15.2 | **0.001** |


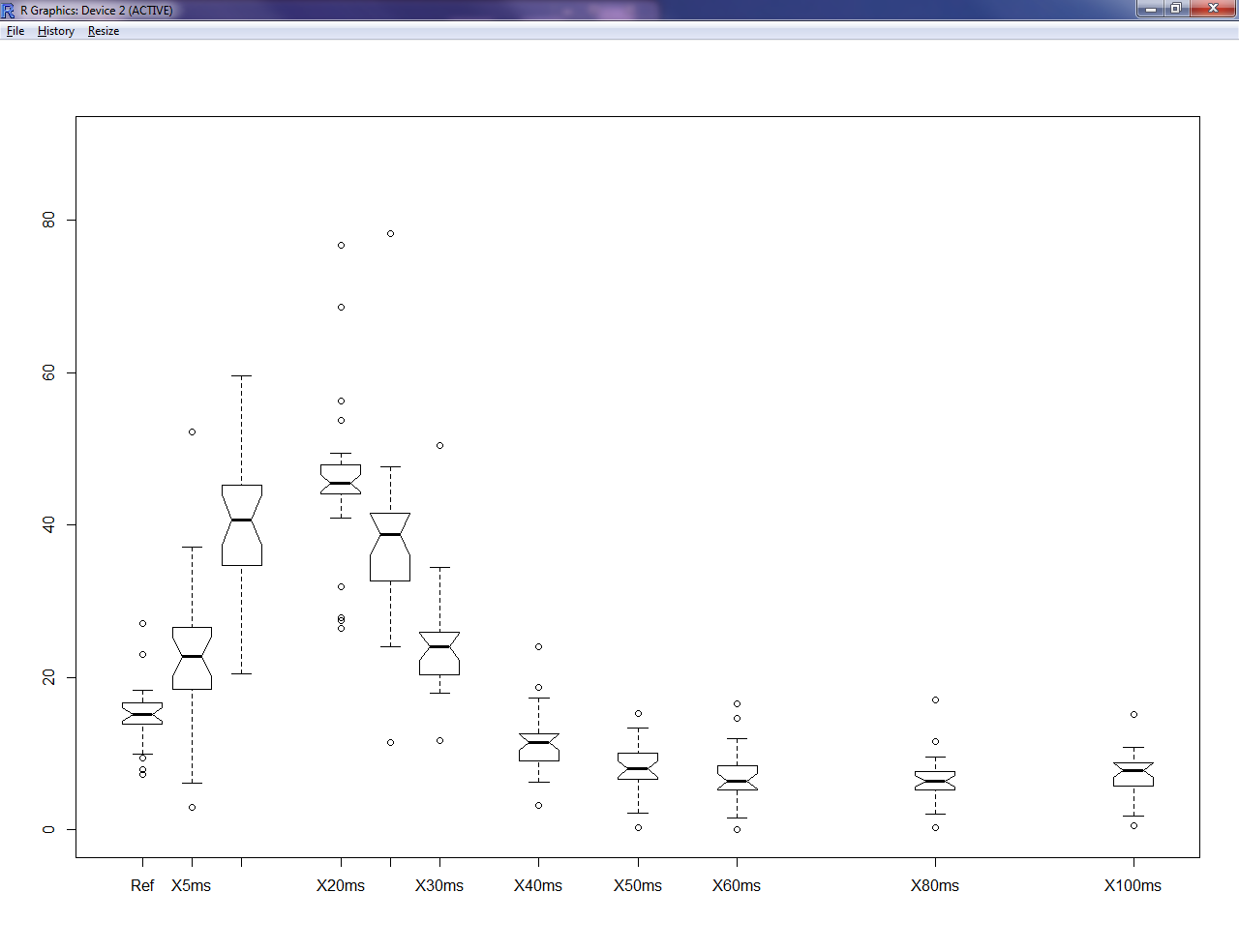


Data for Interval I2

Table I2. Statistical summary for I2, giving an exemplary single trial response (Expl.), the mean and median for all animals tested (n=25); and Wilcoxon Signed Rank test comparing the I2 vs Ref. Bold data are highly significantly different from the reference value.

|  | **Expl.** | **Mean**  **n=25** | **Median**  **n=25** | **SD** | **SEM** | **Min** | **1^st^ Qu.** | **3^rd^ Qu.** | **Max** | **p-value** |
| --- | --- | --- | --- | --- | --- | --- | --- | --- | --- | --- |
| **Ref** | 14.1 | 14.9 | 15.1 | 4.2 | 0.8 | 7.3 | 13.9 | 16.7 | 27.1 | - |
| **5** | 14.4 | 22.3 | 22.4 | 7.7 | 1.5 | 9.2 | 16.3 | 25.6 | 39.5 | **0.001** |
| **10** | 47.6 | 35.8 | 33.8 | 9.8 | 2.0 | 11.7 | 31.9 | 42.8 | 50.5 | **0.001** |
| **20** | 52.7 | 42.7 | 43.4 | 9.5 | 1.9 | 23.9 | 39.6 | 47.2 | 65.0 | **0.001** |
| **25** | 58.1 | 39.0 | 40.7 | 7.4 | 1.5 | 20.3 | 37.2 | 43.7 | 52.0 | **0.001** |
| **30** | 47.1 | 29.1 | 30.4 | 6.1 | 1.2 | 16.9 | 26.0 | 32.6 | 43.9 | **0.001** |
| **40** | 29.8 | 17.4 | 18.2 | 6.0 | 1.2 | 4.1 | 13.0 | 20.3 | 28.6 | 0.03 |
| **50** | 22.0 | 12.7 | 14.5 | 6.7 | 1.3 | 0.8 | 7.1 | 16.3 | 26.4 | 0.09 |
| **60** | 21.4 | 13.8 | 13.5 | 8.1 | 1.6 | 0.0 | 7.0 | 19.4 | 27.9 | 1 n.s. |
| **80** | 17.2 | 12.7 | 13.0 | 6.6 | 1.3 | 3.0 | 7.3 | 15.3 | 28.6 | 0.02 |
| **100** | 14.6 | 15.4 | 14.1 | 7.5 | 1.5 | 0.0 | 10.4 | 21.9 | 29.3 | 1 n.s. |


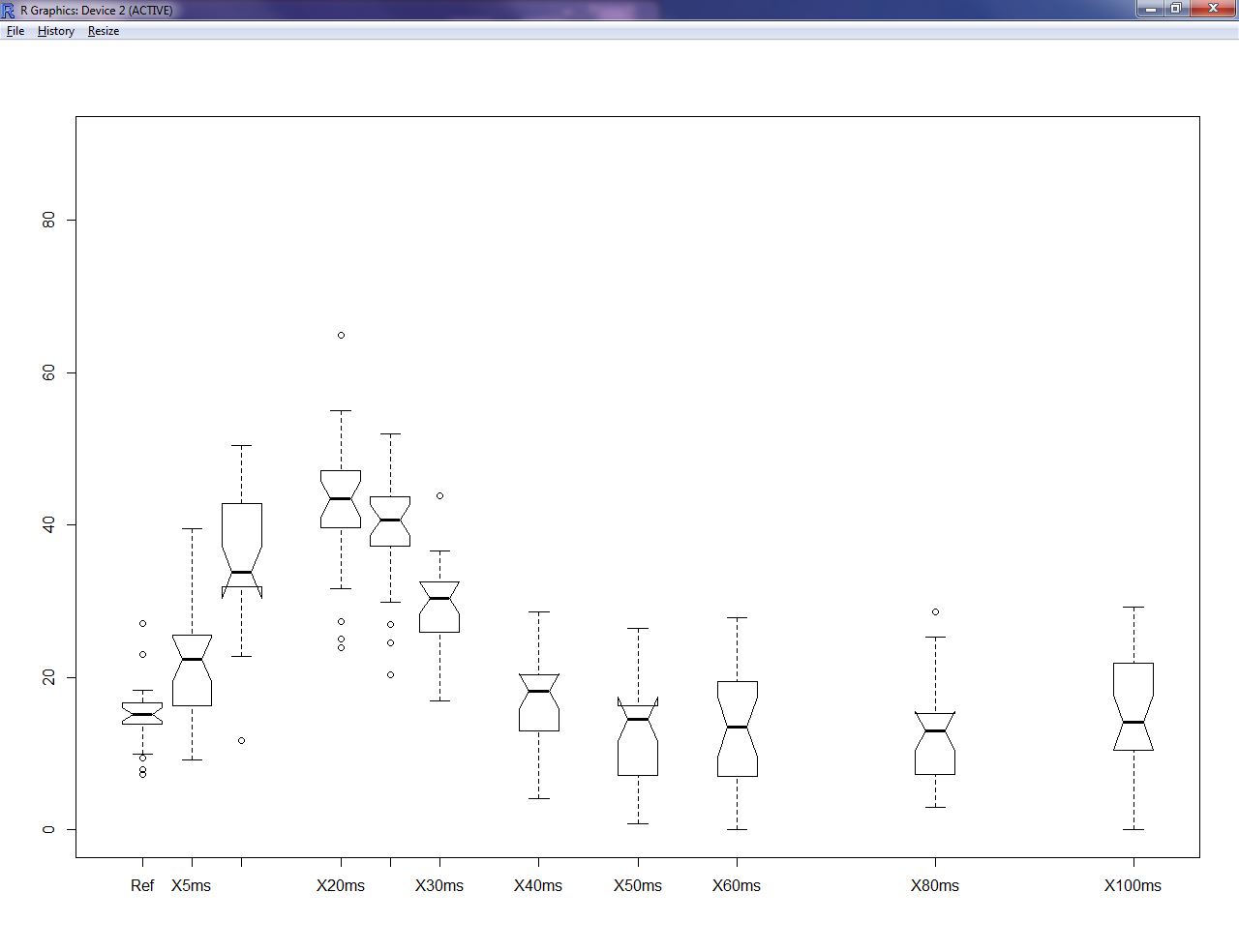


## Data for Pulse P1

Table P1. Statistical summary for P1 giving an exemplary single trial response (Expl.), the mean and median for all animals tested (n=25), and Wilcoxon Signed Rank test comparing the P1 vs Ref. Bold data are highly significantly different from the reference value.

|  | **Expl.** | **Mean**  **n=25** | **Median**  **n=25** | **SD** | **SEM** | **Min** | **1^st^ Qu.** | **3^rd^ Qu.** | **Max** | **p-value** |
| --- | --- | --- | --- | --- | --- | --- | --- | --- | --- | --- |
| **Ref** | 24.3 | 14.9 | 15.1 | 4.2 | 0.8 | 7.3 | 13.9 | 16.7 | 27.1 | - |
| **5** | 85.3 | 46.3 | 45.2 | 11.5 | 2.3 | 23.5 | 38.4 | 54.2 | 68.7 | **0.001** |
| **10** | 90.8 | 49.5 | 50.2 | 11.0 | 2.2 | 29.4 | 43.1 | 56.3 | 76.0 | **0.001** |
| **20** | 84.1 | 46.5 | 44.4 | 10.6 | 2.1 | 28.1 | 39.5 | 55.2 | 64.7 | **0.001** |
| **25** | 51.6 | 33.8 | 32.3 | 9.9 | 2.0 | 8.3 | 29.8 | 39.2 | 57.1 | **0.001** |
| **30** | 32.5 | 21.5 | 19.2 | 9.6 | 1.9 | 4.4 | 15.7 | 23.4 | 49.3 | **0.001**· |
| **40** | 20.9 | 17.6 | 16.0 | 11.0 | 2.2 | 0.0 | 10.8 | 20.4 | 49.2 | 1 n.s. |
| **50** | 18.5 | 14.1 | 13.6 | 9.3 | 1.9 | 2.4 | 7.3 | 16.2 | 36.8 | 1 n.s. |
| **60** | 11.4 | 6.8 | 6.1 | 6.6 | 1.3 | 0.0 | 1.0 | 10.3 | 24.3 | **0.001** |
| **80** | 18.3 | 6.6 | 6.6 | 4.6 | 0.9 | 0.1 | 2.7 | 8.5 | 17.2 | **0.001** |
| **100** | 12.1 | 4.6 | 2.4 | 5.1 | 1.0 | 0.0 | 0.5 | 8.0 | 15.0 | **0.001** |


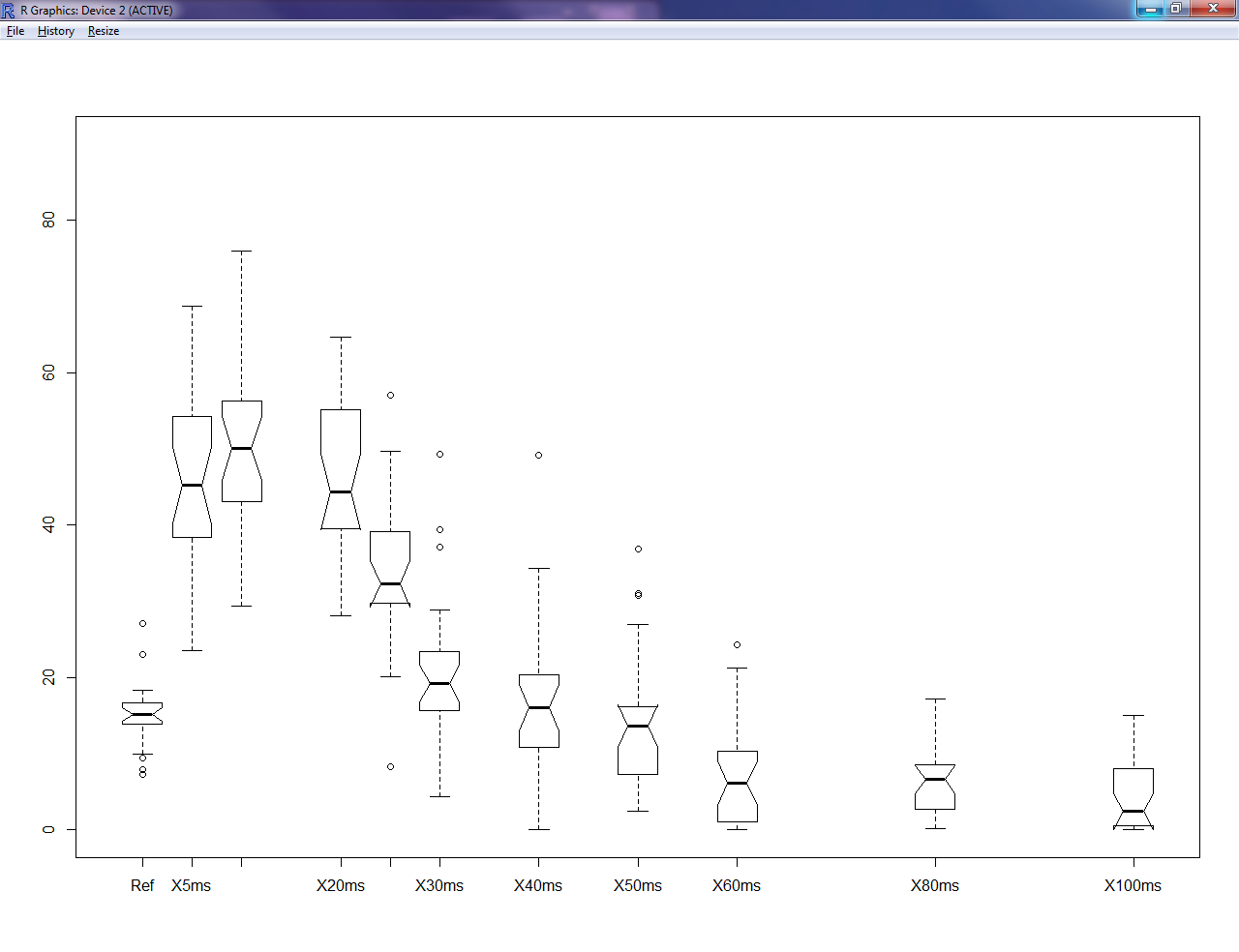


## Data for Pulse P2

Table P2. Statistical summary for P2 giving an exemplary single trial response (Expl.), the mean and median for all animals tested (n=25), and Wilcoxon Signed Rank test comparing the P2 vs Ref. Bold data are highly significantly different from the reference value.

|  | **Expl.** | **Mean**  **n=25** | **Median**  **n=25** | **SD** | **SEM** | **Min** | **1^st^ Qu.** | **3^rd^ Qu.** | **Max** | **p-value** |
| --- | --- | --- | --- | --- | --- | --- | --- | --- | --- | --- |
| **Ref** | 17.9 | 14.9 | 15.1 | 4.2 | 0.8 | 7.3 | 13.9 | 16.7 | 27.1 | - |
| **5** | 27.6 | 21.3 | 20.5 | 9.0 | 1.8 | 6.9 | 12.9 | 27.1 | 41.5 | **0.001** |
| **10** | 63.4 | 45.5 | 44.0 | 8.3 | 1.7 | 31.4 | 38.6 | 50.5 | 63.0 | **0.001** |
| **20** | 72.7 | 45.0 | 43.6 | 11.6 | 2.3 | 29.9 | 34.9 | 53.0 | 70.3 | **0.001** |
| **25** | 54.4 | 38.0 | 37.5 | 10.6 | 2.1 | 22.3 | 30.6 | 45.6 | 57.6 | **0.001** |
| **30** | 30.9 | 26.7 | 26.2 | 9.2 | 1.8 | 12.4 | 18.3 | 30.3 | 47.9 | **0.001** |
| **40** | 8.1 | 18.7 | 17.9 | 8.2 | 1.6 | 5.9 | 11.8 | 23.8 | 39.7 | **0.05** |
| **50** | 7.7 | 17.1 | 17.4 | 6.8 | 1.4 | 6.5 | 11.8 | 21.6 | 30.5 | 1 n.s. |
| **60** | -13.1 | 11.0 | 10.2 | 6.5 | 1.3 | 2.8 | 6.0 | 14.4 | 28.3 | **0.01** |
| **80** | -3.9 | 11.3 | 9.6 | 5.9 | 1.2 | 0.0 | 8.2 | 15.3 | 22.2 | **0.03** |
| **100** | 1.8 | 11.0 | 9.2 | 7.1 | 1.4 | 3.3 | 6.4 | 13.2 | 34.4 | **0.003** |


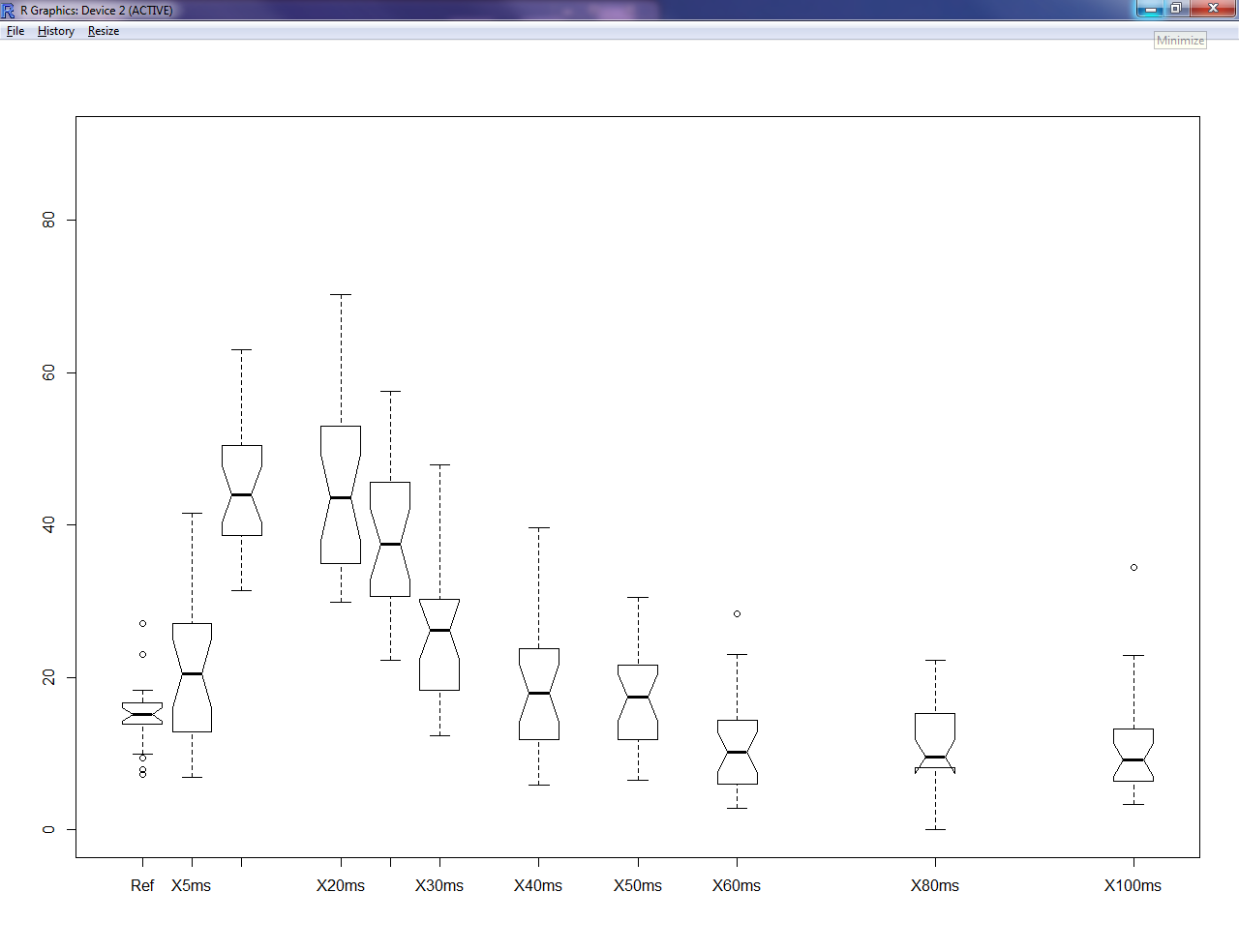


## Data for Pulse P3

Table P3. Statistical summary for P3 giving an exemplary single trial response (Expl.), the mean and median for all animals tested (n=25), and Wilcoxon Signed Rank test comparing the P3 vs Ref. Bold data are highly significantly different from the reference value.

|  | **Expl.** | **Mean**  **n=25** | **Median**  **n=25** | **SD** | **SEM** | **Min** | **1^st^ Qu.** | **3^rd^ Qu.** | **Max** | **p-value** |
| --- | --- | --- | --- | --- | --- | --- | --- | --- | --- | --- |
| **Ref** | 26.2 | 14.9 | 15.1 | 4.2 | 0.8 | 7.3 | 13.9 | 16.7 | 27.1 | - |
| **5** | 33.2 | 21.6 | 21.3 | 9.8 | 2.0 | 5.9 | 16.2 | 25.0 | 49.8 | **0.001** |
| **10** | 42.6 | 31.5 | 32.9 | 11.1 | 2.2 | 11.6 | 25.9 | 37.1 | 53.1 | **0.001** |
| **20** | 48.7 | 41.4 | 43.0 | 11.3 | 2.3 | 19.7 | 36.3 | 46.7 | 67.0 | **0.001** |
| **25** | 46.7 | 42.6 | 45.5 | 13.3 | 2.7 | 11.7 | 35.7 | 52.7 | 66.9 | **0.001** |
| **30** | 50.7 | 40.3 | 44.1 | 13.3 | 2.7 | 1.0 | 35.4 | 48.8 | 60.3 | **0.001** |
| **40** | 36.3 | 39.6 | 42.4 | 10.0 | 2.0 | 17.9 | 35.9 | 45.3 | 54.2 | **0.001** |
| **50** | 38.0 | 35.9 | 36.6 | 10.5 | 2.1 | 10.5 | 34.2 | 42.4 | 53.7 | **0.001** |
| **60** | 45.3 | 32.9 | 33.3 | 8.5 | 1.7 | 10.1 | 31.0 | 38.0 | 46.3 | **0.001** |
| **80** | 28.0 | 34.8 | 34.2 | 8.2 | 1.6 | 18.7 | 33.2 | 38.0 | 58.8 | **0.001** |
| **100** | 26.1 | 31.3 | 30.5 | 10.5 | 2.1 | 9.4 | 28.0 | 34.8 | 66.4 | **0.001** |


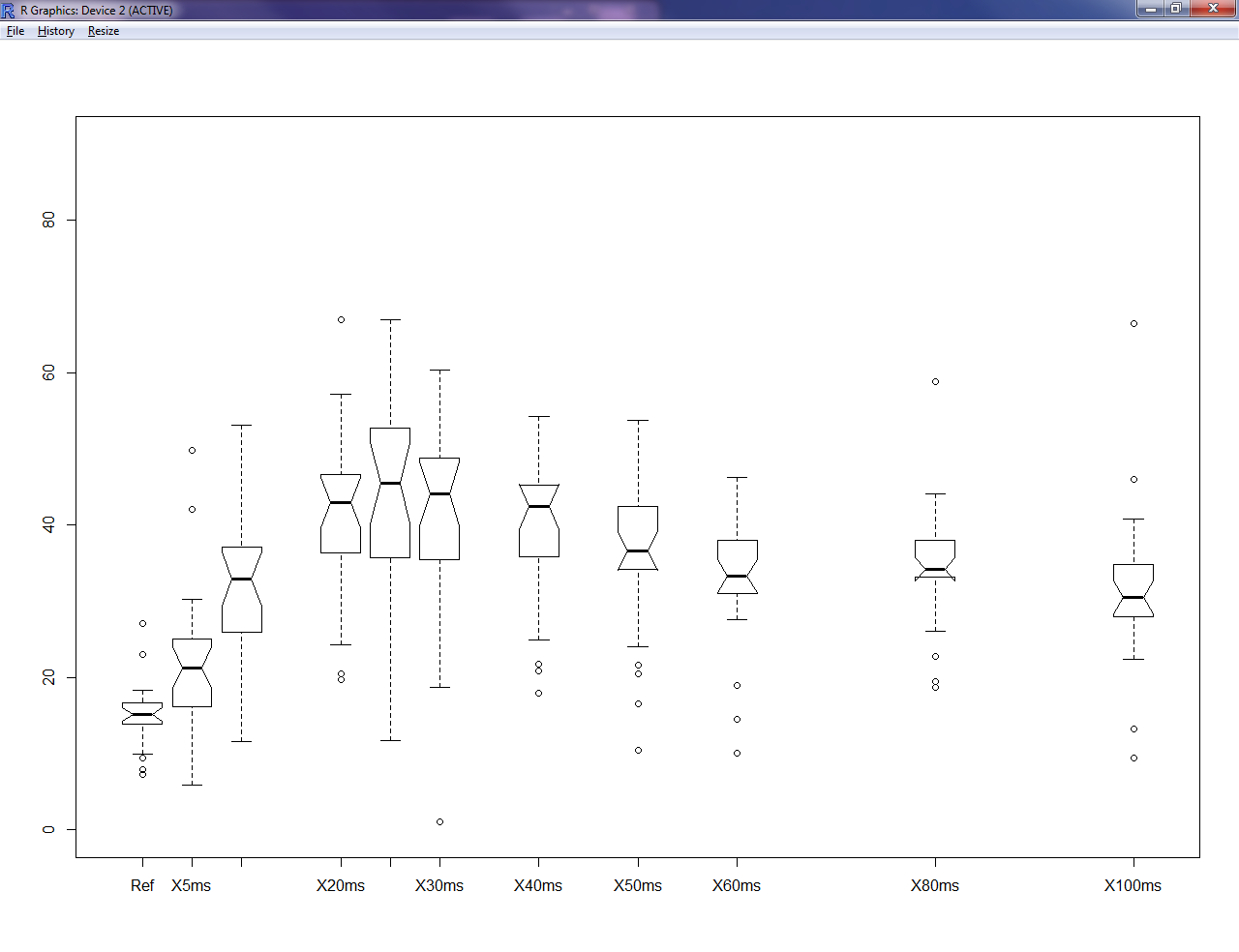


## Data for Attractive and Non-attractive Chirps

Table for Attractive and Non-attractive tests, giving the response of an exemplary single trial (Expl.), the mean and median for all animals tested (n=25), and Wilcoxon Signed Rank test comparing the test data vs Ref. Bold data are highly significantly different to the reference value.

|  | **Expl.** | **Mean**  **n=25** | **Median**  **n=25** | **SD** | **SEM** | **Min** | **1^st^ Qu.** | **3^rd^ Qu.** | **Max** | **p-value** |
| --- | --- | --- | --- | --- | --- | --- | --- | --- | --- | --- |
| **Ref** | 27.0 | 14.9 | 15.1 | 4.2 | 0.8 | 7.3 | 13.9 | 16.7 | 27.1 | - |
| **50*20*5** | 3.0 | 7.9 | 8.1 | 3.8 | 0.8 | -0.6 | 5.7 | 10.3 | 13.6 | **0.001** |
| **5*20*50** | 46.1 | 34.7 | 33.0 | 7.0 | 1.4 | 26.3 | 29.7 | 36.0 | 55.3 | **0.001** |
| **20*20*20** | 47.6 | 48.8 | 46.7 | 9.3 | 1.9 | 38.2 | 42.3 | 55.5 | 72.9 | **0.001** |


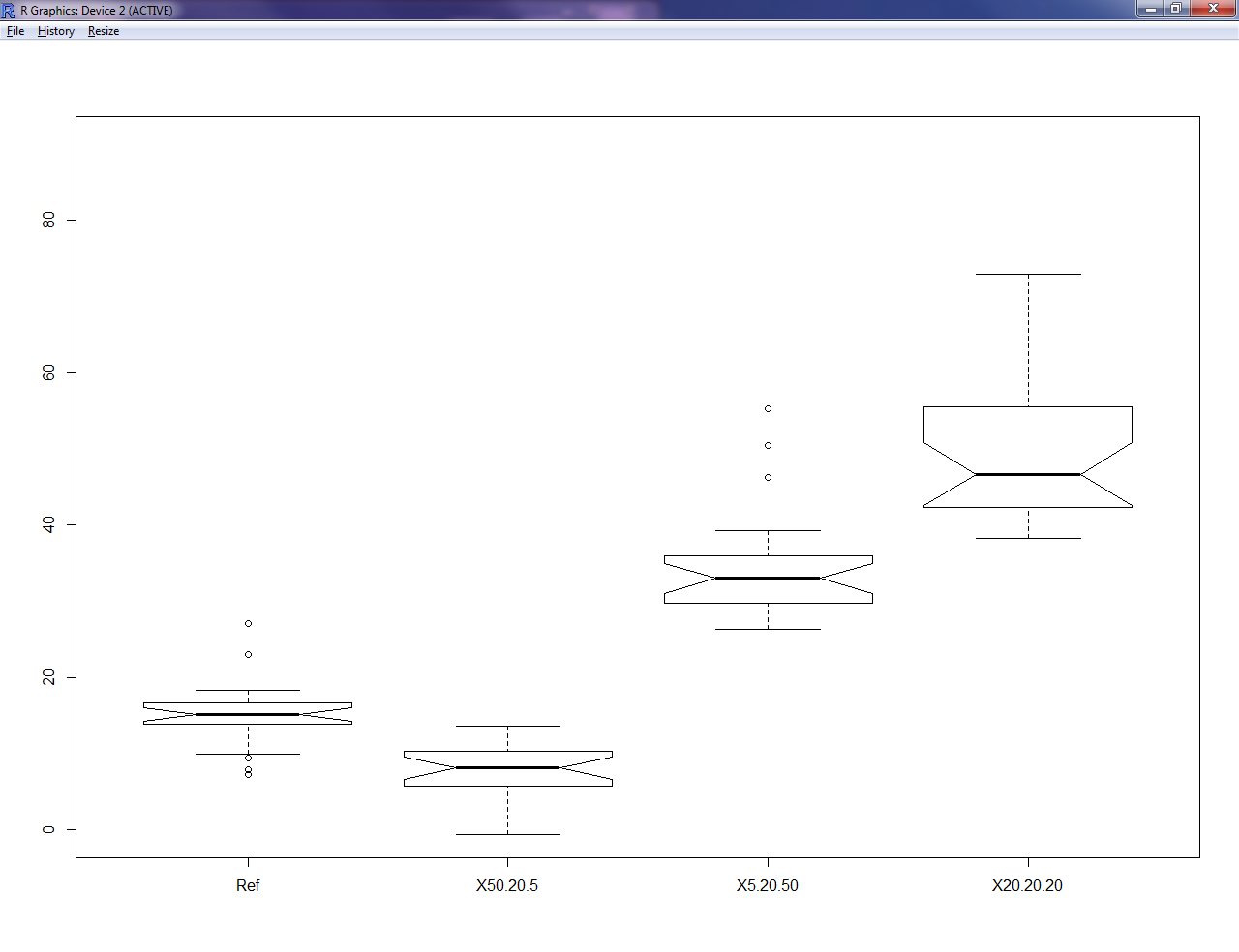

Supplement: Song pattern recognition in crickets based on a delay-line and coincidence-detector mechanism [file rspb20170745supp1.docx]
